# Mortality Burden and Life-Years Lost Across the Age Spectrum for Adults Living with CKD

**DOI:** 10.34067/KID.0000000000000097

**Published:** 2023-03-15

**Authors:** Alexander J. Kula, David K. Prince, Ronit Katz, Nisha Bansal

**Affiliations:** 1 Division of Pediatric Nephrology, Ann & Robert H. Lurie Children's Hospital of Chicago, Chicago, Illinois; 2 Division of Nephrology, Kidney Research Institute, University of Washington, Seattle, Washington; 3 Department of Obstetrics and Gynecology, University of Washington, Seattle, Washington

**Keywords:** CKD, cardiovascular disease, epidemiology, life-years lost, lifespan research

## Abstract

**Key Points:**

- Limited data exist to inform younger persons with CKD how their risk for mortality compares with equivalently aged individuals without CKD.
- Compared with the general population without CKD, the age-stratified risk for mortality was highest in younger individuals with CKD.
- From a lifetime perspective, the estimated reduction of lifespan secondary to CKD was greatest at younger ages.

**Background:**

Younger individuals living with CKD face a lifetime at risk for complications and mortality. Limited data exist to inform individual patients with CKD across the lifespan how their risk for mortality compares with equivalently aged individuals without CKD, particularly at younger ages. The objective of this study was to provide age-specific contexts to the risk of mortality associated with a diagnosis of CKD.

**Methods:**

We created a pooled study cohort using participants with CKD enrolled in the Chronic Renal Insufficiency Cohort along with participants aged 21–75 years included in the 1999–2008 National Health and Nutrition Examination Survey surveys. Age-stratified mortality rates, along with unadjusted and adjusted hazard ratios (HRs) for mortality, were generated to compare differences between those with and without CKD. The mean life-years lost (LYL) relating to CKD was calculated using Centers for Disease Control and Prevention life tables.

**Results:**

A total of 16,725 participants were included. Mortality rates were higher in those with CKD at all ages. The adjusted age-stratified HR for mortality in those with CKD versus without was highest in the 21–35 years strata (HR [95% confidence interval (CI)], 4.9 [2.8 to 8.6])) and lowest in the 65–75 years strata (HR [95% CI], 2.0 [1.7 to 2.3]). The mean LYL secondary to CKD was inversely related with increasing age.

**Conclusions:**

Compared with age-matched peers without CKD, the age-stratified risk for mortality and LYL associated with a diagnosis of CKD is highest in younger individuals. Further research is needed to elucidate the societal and personal costs of premature mortality associated with CKD in young adults.

## Graphical abstract

**Figure:**
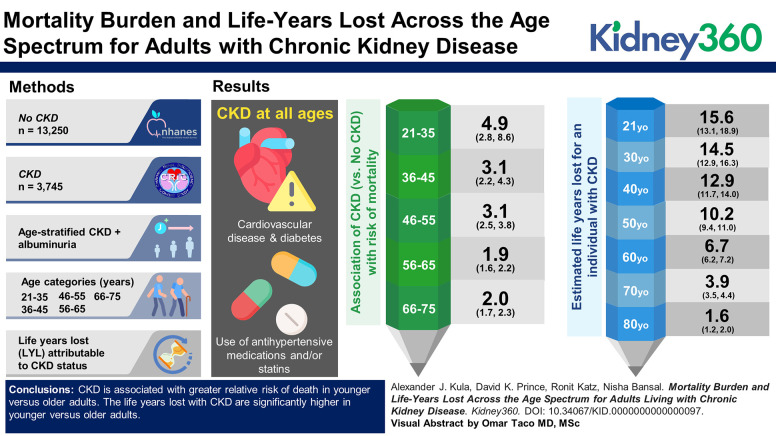

## Introduction

CKD is a disease that can affect individuals of any age, and in most cases, it is irreversible. It is well known that CKD is associated with increased risk for morbidity and mortality. Important differences in this risk of mortality may exist across the age span.

For example, observational studies demonstrate that age-stratified mortality rates are significantly higher in those with a diagnosis of ESKD requiring dialysis compared with the general population.^1,2^ The aged-matched relative difference in mortality rates is highest in younger individuals and subsequently decreases with older age. Less is known on differences in mortality rates across the entire spectrum of adulthood in patients with nondialysis requiring CKD, a much more prevalent condition compared with ESKD. A study of older patients receiving care within the Veterans Administration noted the age-matched risk for mortality between those with and without CKD decreased at older ages.^3^ Applying these findings to the larger American population across a broader age range is challenged by the unique characteristics of the subpopulation receiving care through Veterans Affairs. More information is needed to generalize our understanding of mortality risk associated with CKD for all ages to appropriately provide care for adults with CKD across all ages.

In particular, younger adults with CKD have been underrepresented in previous studies of CKD. Moreover, the number of younger Americans with CKD is liable to grow secondary to increasing rates of adolescent and young adults with obesity, type 2 diabetes, and hypertension.^4–8^ Understanding their mortality risk, and subsequent decrease in estimated lifespan, compared with similarly aged peers without CKD can help define the significance of a CKD diagnosis in this population.

To address our present gaps in understandings, we performed an observational study using a pooled cohort to determine the absolute and relative risks of mortality in patients with versus without CKD, stratified across ages from young to older adults. Furthermore, we calculated life-years lost (LYL) in those with CKD versus the general population at various ages. The goal being to better delineate the risk for premature mortality associated with CKD using a lifespan perspective.

## Methods

### Study Population

To compare CKD with non-CKD patients, our study sample comprised patients with CKD included in the Chronic Renal Insufficiency Cohort (CRIC) and persons from the general population without CKD from the National Health and Nutrition Examination Survey (NHANES). In total, the CRIC study enrolled 3939 participants age 21–75 years between June 2003 and August 2008 at seven clinical centers across the United States. Patients with eGFR of 20–70 ml/min per 1.73 m^2^ using the Modification of Diet in Renal Disease study equation were eligible for enrollment, and patients with New York Heart Association class III or IV heart failure were ineligible. Additional details on study design, inclusion and exclusion criteria, and baseline characteristics of the participants have been previously published.^9,10^ For this study, we excluded all participants whose eGFR at the baseline visit was ≥70 ml/min per 1.73 m^2^ (*n*=193) because we were interested in focusing on those with CKD by the eGFR criteria for the present analysis.

Individuals included in the 1999–2008 NHANES comprised the non-CKD comparison group of our cohort. The Centers for Disease Control along with the National Center for Health Statistics administer and manage the NHANES survey. In brief, NHANES is a cross-sectional study administered and managed by the Centers for Disease Control and Prevention and the National Center for Health Statistics tasked with assessing the health and nutritional status of the US population as a whole. Sampling occurs nationwide and is designed to capture a nationally representative population. Data are collected through home interview, and a number of participants also agree to undergo a standardized physical examination and give blood and urine samples for centralized laboratory testing. We excluded all NHANES participants 21 years or younger or older than 75 years, with missing physical examination or laboratory measurements, or if participants met any criteria for CKD discussed below. Sample weighting was not performed for NHANES data when creating the pooled cohort since a similar weighting was not available for the CRIC study. Therefore, participants included from NHANES represent a large, diverse, and well-characterized population of non-CKD control cases, rather than a precisely representative sample of the US population.

### Exposure: CKD Versus Non-CKD

The primary exposure in this analysis was CKD status*.* All eGFR calculations were made using the original Chronic Kidney Disease Epidemiology Collaboration (CKD-EPI) equation to remain consistent with the CRIC protocol*.* To account for normal age-related declines in GFR, we created an age-stratified CKD definition: For persons aged 21–40 years, eGFR <75 ml/min per 1.73 m^2^ or urine albumin-to-creatinine ratio (UACR) >30 mg/g; for persons aged 40.1–65 years, eGFR <60 ml/min per 1.73 m^2^ or UACR >30 mg/g; and for persons older than 65 years, eGFR <45 ml/min per 1.73 m^2^ or UACR >30 mg/g. We used the age-stratified eGFR cutoffs recommended previously by Delanaye *et al.* and included an albuminuria criterion to remain consistent with KDIGO definitions.^11,12^ Using this stratified CKD definition has been advocated for when studying persons with CKD across wide age ranges to improve specificity.^11^

### Outcome: Mortality

In the CRIC cohort, mortality was identified through report from next of kin, review of hospital records, retrieval of death certificates or obituaries, and linkage with the Social Security Mortality Master File. Mortality data for NHANES participants were ascertained by linkage to the National Death Index. Mortality follow-up in NHANES lasted until December 31, 2019, and CRIC follow-up lasted until December 31, 2021.

### Covariates

For both NHANES and CRIC participants, demographic, medical history, and medication use data were ascertained by participant self-report. Blood pressure was measured by centrally trained staff using a standardized method in a quiet, standardized setting. The mean of three-seated resting blood pressure readings was used to define the systolic blood pressure (SBP) value.^13,14^ eGFR was calculated using the CKD-EPI equation using serum creatinine values from both studies. Both CRIC and NHANES used centralized measurement of serum and urine laboratory values.

### Statistical Methods

Participants were stratified into the following age categories based off age at NHANES examination or CRIC baseline visit: 21–35 years, 36–45 years, 46–55 years, 56–65 years, and 66–75 years. Participant characteristics were reported as mean and standard deviation for normally distributed variables or as median and interquartile range (IQR) for skewed variables. Baseline characteristics were stratified by CKD status and age strata. Mortality rates were calculated within CKD status and age strata by summing the number of deaths divided by the total observed person time with corresponding exact 95% confidence intervals (CIs). We used Kaplan-Meier estimates to generate survival curves to estimate the probability of survival stratified by baseline CKD status.

We investigated the association between CKD status at baseline with mortality through a series of nested Cox proportional hazards models. Adjusted models included adjustment for age, race/ethnicity, sex, smoking status, and diabetes. Cox models were performed in parallel within each age category. We calculated point estimates for hazards ratios (HRs) and 95% CIs from the Cox models. In Cox models, persons in CRIC were censored at the last date of follow-up. ESKD was not a censoring event for CRIC participants.

LYL attributable to CKD status was calculated across the lifespan using the *Lillies* package from R statistics. This approach compares survival curves by CKD status conditioned on age to estimate the difference between them, corresponding to the estimated LYL.^15^ With this approach, one can estimate LYL from a specific age to some upper age limit. We set this limit as 90 years given we have limited follow-up time in individuals older than 90 years. The reported LYL can be interpretated as the LYL before the age 90 years. CIs were calculated using a nonparametric bootstrap approach.

All statistical analyses were conducted using SPSS Statistics version 27.0 (IBM Corp, Armonk, NY) or R version 3.6.2 (R Core Team, 2019).

## Results

### Baseline Characteristics

In total, this analysis included 16,725 participants: 3745 individuals with CKD and 13,250 individuals without CKD. The age-stratified baseline characteristics of each cohort and combined study population are presented in Table 1. At all age strata, individuals with CKD were more likely to identify as Black and have an education level of some college or greater. The difference in mean SBP between those with and without CKD was greatest in the 21–35 years age strata (mean SBP±SD, CKD: 122±20 mm Hg versus non-CKD: 113±12 mm Hg). The prevalence of cardiovascular disease (CVD) and diabetes was higher in individuals with CKD at all ages. LDL levels were higher in those without CKD at all age stages, with the largest difference in individuals aged 66–75 years (mean LDL±SD, CKD: 99±34 mg/dl versus non-CKD: 123±36 mg/dl). A larger proportion of those with CKD had the use of antihypertensive medications and/or statins.

**Table 1:** Baseline characteristics of study population

Variable	21–35		36–45		46–55		56–65		66–75	
	CKD	Non-CKD	CKD	Non-CKD	CKD	Non-CKD	CKD	Non-CKD	CKD	Non-CKD
	*N*=183	*N*=4523	*N*=321	*N*=2732	*N*=697	*N*=2333	*N*=1411	*N*=1963	*N*=863	*N*=1699
Age (yr), mean±SD	31±4	28±4	41±3	41±3	51±3	50±3	60±3	61±3	70±3	70±3
Male, *n* (%)	95 (52)	1933 (43)	195 (61)	1357 (50)	380 (55)	1183 (51)	793 (56)	970 (49)	472 (55)	858 (51)
**Race/ethnicity**, ***n* (%)**										
Black	70 (38)	868 (19)	129 (40)	571 (21)	338 (48)	472 (20)	649 (46)	336 (17)	372 (43)	269 (16)
Hispanic	36 (20)	1438 (32)	53 (17)	813 (30)	106 (15)	500 (21)	161 (11)	548 (28)	101 (12)	405 (24)
Other	10 (5)	196 (4)	20 (6)	108 (4)	21 (3)	93 (4)	54 (4)	69 (4)	25 (3)	38 (2)
White	67 (37)	2021 (45)	119 (37)	1240 (45)	232 (33)	1268 (54)	547 (39)	1010 (51)	365 (42)	987 (58)
**Education level, *n* (%)**										
Less than high school	23 (13)	1158 (26)	47 (15)	717 (26)	150 (22)	539 (23)	323 (23)	683 (35)	245 (28)	650 (38)
High school graduate	32 (17)	1075 (24)	60 (19)	617 (23)	127 (18)	560 (24)	262 (19)	430 (22)	192 (22)	424 (25)
Some college or greater	128 (70)	2286 (51)	214 (67)	1396 (51)	420 (60)	1232 (53)	826 (59)	848 (43)	426 (49)	620 (36)
Missing	0 (0)	4 (0)	0 (0)	2 (0)	0 (0)	2 (0)	0 (0)	2 (0)	0 (0)	5 (0)
**Prevalent CVD, *n* (%)**										
MI or CHD	5 (3)	15 (0)	11 (3)	37 (1)	100 (14)	79 (3)	311 (22)	168 (9)	233 (27)	207 (12)
Heart failure	5 (3)	7 (0)	17 (5)	14 (1)	69 (10)	45 (2)	164 (12)	62 (3)	107 (12)	73 (4)
Stroke	4 (2)	15 (0)	11 (3)	24 (1)	71 (10)	43 (2)	173 (12)	60 (3)	118 (14)	80 (5)
**CV risk factors**										
Current or former smoker, *n* (%)	56 (31)	1789 (40)	124 (39)	1293 (47)	387 (56)	1262 (54)	849 (60)	1116 (57)	512 (59)	911 (54)
Diabetes, *n* (%)	50 (27)	49 (1)	128 (40)	101 (4)	361 (52)	179 (8)	780 (55)	269 (14)	475 (55)	255 (15)
Triglycerides (mg/dl), mean±SD	173±165	133±104	161±101	147±136	170±125	156±133	161±126	161±165	149±98	153±83
LDL (mg/dl), mean±SD	113±43	113±35	109±38	121±34	104±36	127±34	101±35	128±37	99±34	123±36
**CV measures, mean±SD**										
SBP (mm Hg)	122±20	113±12	122±22	118±14	129±23	124±16	130±22	132±19	132±23	137±21
DBP (mm Hg)	78±13	67±11	77±13	74±11	76±13	76±10	71±12	73±11	66±12	70±12
BMI (kg/m^2^)	31±9	28±7	32±9	29±6	33±9	29±6	33±8	29±6	32±7	29±5
**Medications, *n* (%)**										
Antihypertensive	152 (83)	74 (2)	280 (87)	223 (8)	640 (92)	526 (23)	1334 (95)	766 (39)	842 (98)	901 (53)
Statin	48 (26)	10 (0)	129 (40)	70 (3)	347 (50)	203 (9)	862 (61)	353 (18)	552 (64)	457 (27)
**Kidney function, mean±SD**										
eGFR (ml/min per 1.73 m^2^)	45±17	116±16	43±16	105±14	40±13	96±14	40±13	90±13	34±9	81±15
UACR (mg/g)	1386±2271	7±5	1056±1969	7±5	1021±2203	7±5	584±1382	9±6	469±1221	9± 6

CVD, cardiovascular disease; MI, myocardial infarction; CHD, coronary heart disease; CV, cardiovascular; SBP, systolic blood pressure; DBP, diastolic blood pressure; BMI, body mass index; UACR, urine albumin-to-creatinine ratio.

### Mortality Rates Across Age Strata

**Figure 1:**
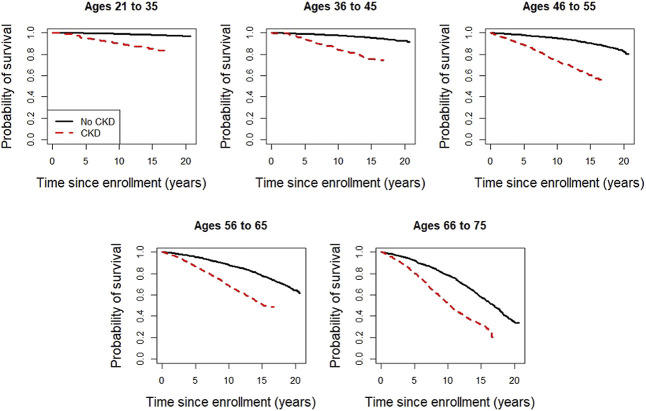
Kaplan-Meier plots stratified by CKD status depicting probability of survival from the time of enrollment across different age strata.

**Figure 2:**
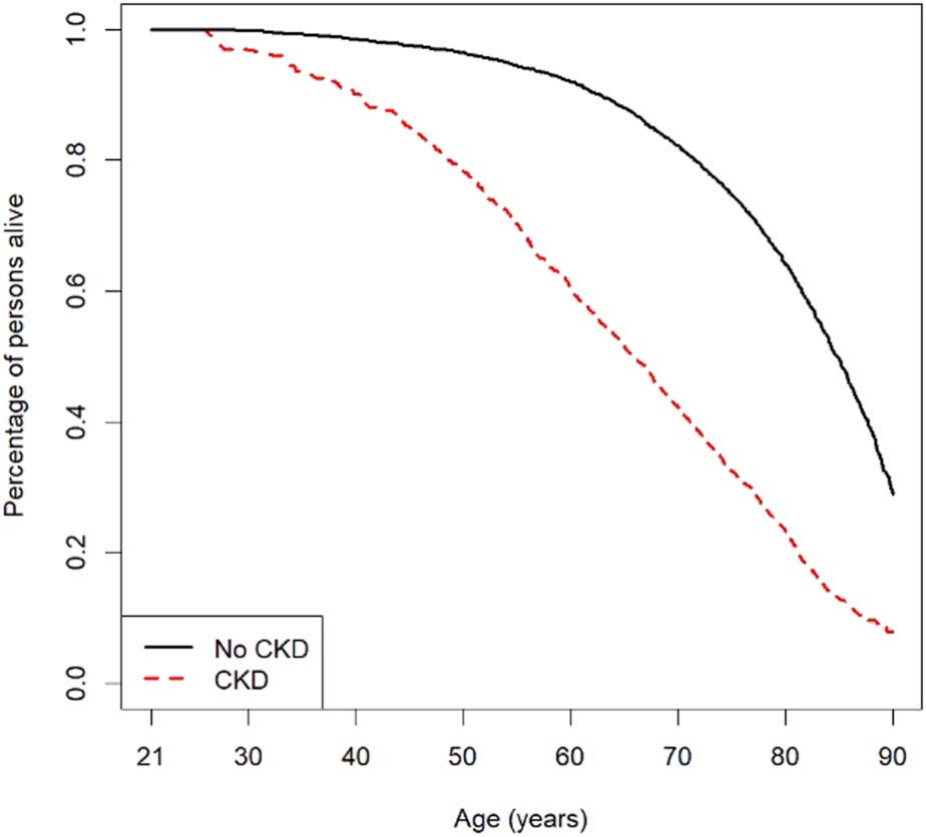
Human survival curves for individuals with CKD enrolled in the CRIC study and without CKD enrolled in the 1999–2008 NHANES.

Over the study period, 1544 (41%) individuals with CKD died, while 1962 (15%) of those without CKD died. The median (IQR) follow-up time for the total cohort, CKD subgroup, and non-CKD subgroup was 15.6 (13.6–17.9), 13.1 (7.1–15.0), and 16.4 (14.2–18.5) years, respectively. There was a graded increase in the mortality rate with advancing age (Table 2 and Figure 1). Mortality rates in individuals with CKD increased from 10.7 deaths per 1000 person-years (95% CI, 7.0 to 15.6) in those aged 21–35 years to 67.7 deaths per 1000 person-years (95% CI, 62.1 to 73.7) in the oldest age category (66–75 years). Comparably, the mortality rate for those without CKD aged 21–35 years and 66–75 years was 1.3 (95% CI, 1.1 to 1.6) and 37.9 (95% CI, 35.4 to 40.5) per 1000 person-years, respectively. Mortality rates were higher in study participants with CKD compared with those without CKD in all age groups (Table 2 and Figure 1). Kaplan-Meier curves for each age group, stratified by CKD status, are presented in Figure 2.

**Table 2:** Incidence rates (IR per 1000 person-years) for mortality by age and CKD status

Age Group	CKD		Non-CKD	
	Event Count	IR (95% CI)	Event Count	IR (95% CI)
21–35	26	10.7 (7.0 to 15.6)	101	1.3 (1.1 to 1.6)
36–45	74	17.8 (14.0 to 22.4)	159	3.5 (3.0 to 4.1)
46–55	260	32.4 (28.6 to 36.6)	291	7.8 (6.9 to 8.7)
56–65	641	41.1 (38.0 to 44.4)	541	18.1 (16.6 to 19.7)
66–75	543	67.7 (62.1 to 73.7)	870	37.9 (35.4 to 40.5)

IR: incidence rate per 1000 person-years; CI, confidence interval.

### Association of CKD Status and Mortality by Age Strata

Compared with those without CKD, the risk for mortality associated with CKD was significantly higher in all age strata (Table 3). However, the magnitude of the association of CKD with mortality decreased with increasing age. In unadjusted models, CKD status was associated with a HR (95% CI) of 8.8 (5.7 to 13.6) and 2.3 (2.0 to 2.5) within the 21–35 years and 66–75 years age group, respectively. In fully adjusted models, the HR was highest in the youngest age group (HR [95% CI] for mortality for CKD versus non-CKD: 4.9 [2.8 to 8.6]) and lowest in the oldest participants (adjusted HR [95% CI] for mortality for CKD versus non-CKD: 2.0 [1.7 to 2.3])

**Table 3:** Association of CKD (versus non-CKD) with risk of mortality, stratified by age

Age Group	Total Number		Unadjusted	Adjusted
	CKD	Non-CKD	HR (95% CI)	HR (95% CI)
21–35	183	4523	8.8 (5.7 to 13.6)	4.9 (2.8 to 8.6)
36–45	321	2732	5.9 (4.4 to 7.8)	3.1 (2.2 to 4.3)
46–55	697	2333	5.0 (4.2 to 5.9)	3.1 (2.5 to 3.8)
56–65	1411	1963	2.7 (2.4 to 3.0)	1.9 (1.6 to 2.2)
66–75	863	1699	2.3 (2.0 to 2.5)	2.0 (1.7 to 2.3)

HR, hazard ratio; CI, confidence interval.

Adjusted: age, race/ethnicity, sex, smoking status, and diabetes.

### LYL in Patients with CKD Across Age Strata

The total LYL for those with CKD compared with individual without CKD was inversely related to age (Table 4). On average, a 20-year-old individual with CKD can expect 15.6 LYL compared with a similarly aged individual without CKD. In comparison, a 70-year-old individual with CKD would on average lose 3.9 life years. Figure 2 presents the results of this modeling in the form of survival curves.

**Table 4:** Estimated life-years lost for an individual with CKD compared with a similarly aged individual without CKD

Age	LYL (95% CI) Due to CKD
21 yrs	15.6 (13.1 to 18.9)
30 yrs	14.5 (12.9 to 16.3)
40 yrs	12.9 (11.7 to 14.0)
50 yrs	10.2 (9.4 to 11.0)
60 yrs	6.7 (6.2 to 7.2)
70 yrs	3.9 (3.5 to 4.4)
80 yrs	1.6 (1.2 to 2.0)

LYL, life-years lost; CI, confidence interval.

## Discussion

This observational study of 16,725 participants with and without CKD aged 21–75 years demonstrates that the relative risk of morality associated with CKD is greater at younger ages and is attenuated as patients' age. While the absolute mortality rates were highest in older individuals with and without CKD, young adults with CKD had the highest relative risk increase. This translated to an average of 15.6 LYL for a 21-year-old individual with CKD compared with a similarly aged individual without CKD. The results from this study improve our understanding of the lifetime implications accompanying a diagnosis of CKD across the age span. These data also highlight the need for enhanced medical attention to younger adults with CKD, in whom therapies to reduce mortality may have substantial effect over their lifetime.

Our results contribute a new dimension to previous research describing the risk for, and burden of, mortality related to a diagnosis of CKD. Using clinical data from adult patients of the Kaiser Permanente system, Go *et al.* clearly illustrated that CKD was associated with an increased risk for mortality.^16^ A more recent study by the Global Burden of Disease CKD consortia (GBD-CKD) demonstrated a worldwide increase in the prevalence of CKD and that CKD was now the 12th leading cause of death worldwide. The GBD-CKD study found that CKD across the globe was associated with 28.5 million LYL in 2017 alone.^17^ Similar from the observations of our analysis, a study of adults 60 years or older with hypertension in Hong Kong generally illustrated decreasing LYL associated with CKD with older age.^18^ However, this study did not include any observations in individuals younger than 60 years, included only individuals with hypertension, and did not explicitly describe the independent, age-stratified effect of CKD in LYL. Our manuscript builds on the previous observation by Hallan *et al.* of an inverse relationship between older age and mortality risk associated with CKD.^19^ Specifically, we aimed to provide greater granularity for age-associated risks in persons younger than 55 years and translate age-specific risks to estimations of LYL. Given the growing population of younger adults with CKD, our data may provide additional insight on lifetime risks associated with CKD.

The significance of our findings may differ depending on the relative importance placed on absolute versus relative differences in mortality rates.^20^ For the individual with CKD, interpreting the significance of relative versus absolute risk as it pertains to themselves may be nebulous.^21,22^ We see including LYL in discussions of risk as a less abstract way to frame the consequence of CKD at various ages. Using this framework, the presence of CKD implies a much larger reduction in lifespan at younger ages compared with older ages. While younger persons with CKD may have the greatest gain in lifespan with improved CKD care, this population has poor representation in therapeutic trials. We see the results of our study suggesting that young adults with CKD deserve special focus in future research because they are at greatest risk for LYL related to their CKD.

Previous work suggests CVD as the primary contributor to increased mortality in individuals with CKD at all ages.^16,23,24^ In our study, the greatest imbalance in cardiovascular risk factors between those with and without CKD was in the 21–35 years age strata. The cardiovascular phenotype of young adults with CKD seemed similar to healthy controls aged 20–30 years. There is an emerging viewpoint that CKD could be framed as a problem of premature aging.^25^ As such, there may be an opportunity to apply evidence-based strategies to reduce the risk of mortality (*e.g.*, reducing blood pressure) as well as develop new, cardiovascular interventions specific to young adults with CKD.

Relevant strengths of this analysis included deriving a study population from high-quality data sources. CKD participants came from a well-characterized cohort. Although we did not perform weighting of NHANES data, we were able to use a large sample of well-described individuals with broad geographic, biologic, and socioeconomic diversity. Taken together, this allowed us to make comparisons of mortality rates and risk across a wide range of ages in an exclusively American study population. Many of the insights generated by this analysis were made possible by creating a pooled study population. The modest population size of young adults with known CKD makes undertaking research projects at a geographical area or medical center challenging. Studies of CKD in pediatrics have demonstrated the potential of nationwide collaboration to enroll a sizable study population.^26^ Until this can be realized for young adults with CKD, pooling data from multiple sources may be required. Advantages of this methodology include reduced costs, increased sample size, and improved geographic diversity.^27^

Limitations include that CRIC participants were recruited from and closely followed in CKD clinics, making them less generalizable to the American CKD population. We could also not identify the duration of CKD at the time of enrollment in CRIC. Owing to the need to harmonize two datasets, we were not able to include variables that were not collected in both or variables for which the measurement used a nonshared protocol or scale. Owing to missing data in CRIC and NHANES, we could not include analyses on cause of death. Further research is needed, especially how risk for cardiovascular mortality between those and without CKD changes with age. Some individuals included from NHANES could have developed CKD after the baseline visit. We attempted to minimize confounding by using potentially relevant covariables; however, as with all observational studies, we could not eliminate the risk for unmeasured confounding.

In conclusion, CKD is associated with greater relative risk of death in younger versus older adults. The LYL with CKD is significantly higher in younger versus older adults. Further research is required to elucidate the societal and personal costs of premature mortality in a younger population with many productive and meaningful years left ahead. Our hope is these results will inform the development of new strategies to identify and treat young individuals living with CKD in this country.

## Data Availability

Previously published data were used for this study. JASN Jul 2003, 14 (suppl 2) S148-S153; DOI: 10.1097/01.ASN.0000070149.78399.CE.
